# Bionic eye system mimicking microfluidic structure and intraocular pressure for glaucoma surgery training

**DOI:** 10.1371/journal.pone.0271171

**Published:** 2022-07-11

**Authors:** Toshiro Yamanaka, Tomonori Niino, Seiji Omata, Kanako Harada, Mamoru Mitsuishi, Koichiro Sugimoto, Takashi Ueta, Kiyohito Totsuka, Tomoyasu Shiraya, Fumiyuki Araki, Muneyuki Takao, Makoto Aihara, Fumihito Arai

**Affiliations:** 1 Department of Mechanical Engineering, School of Engineering, The University of Tokyo, Bunkyo, Tokyo, Japan; 2 NIDEK Co., Ltd., Gamagori, Aichi, Japan; 3 Faculty of Advanced Science and Technology, Kumamoto University, Kumamoto, Japan; 4 Center for Disease Biology and Integrative Medicine (CDBIM), The University of Tokyo, Bunkyo, Tokyo, Japan; 5 Department of Ophthalmology, School of Medicine, The University of Tokyo, Bunkyo, Tokyo, Japan; LV Prasad Eye Institute, INDIA

## Abstract

Among increasing eye diseases, glaucoma may hurt the optic nerves and lead to vision loss, the treatment of which is to reduce intraocular pressure (IOP). In this research, we introduce a new concept of the surgery simulator for Minimally Invasive Glaucoma Surgery (MIGS). The concept is comprised of an anterior eye model and a fluidic circulatory system. The model made of flexible material includes a channel like the Schlemm’s canal (SC) and a membrane like the trabecular meshwork (TM) covering the SC. The system can monitor IOP in the model by a pressure sensor. In one of the MIGS procedures, the TM is cleaved to reduce the IOP. Using the simulator, ophthalmologists can practice the procedure and measure the IOP. First, considering the characteristics of human eyes, we defined requirements and target performances for the simulator. Next, we designed and manufactured the prototype. Using the prototype, we measured the IOP change before and after cleaving the TM. Finally, we demonstrated the availability by comparing experimental results and target performances. This simulator is also expected to be used for evaluations and developments of new MIGS instruments and ophthalmic surgery robots in addition to the surgical training of ophthalmologists.

## Introduction

Eye diseases are worldwidely increasing as the number of elderly people aged 65 and over increases. Among those diseases, glaucoma is one of leading causes of vision impairment [1]. In 2013, the global prevalence of glaucoma was estimated as 3.54% for the adult population aged 40–80 years [2]. The number of people with glaucoma worldwide was estimated as 64.3 million in 2013 and is assumed to increase to 111.8 million in 2040 [2].

Glaucoma may hurt the optic nerves and lead to vision loss, the treatment of which is to reduce intraocular pressure (IOP). Minimally invasive glaucoma surgery (MIGS) is one of the commonly used methods to treat primary open-angle glaucoma as it reduces the IOP. Trabeculotomy ab interno procedure relieves the hydraulic resistance to aqueous flow by cleaving the trabecular meshwork (TM) and the inner walls of Schlemm’s canal (SC) [3].

Ophthalmologists have to train surgical procedures before they are performed on patients, so artificial [4] or animal models are required for the training. As well as the bioethical issues, animal models have a number of issues, such as different anatomical properties from those of humans, stable procurement, characteristic guarantee, and so on. Therefore artificial simulators are considered ideal solutions [5]. Developing simulators using virtual reality (VR) is a significant challenge [6, 7]. However, compared to the VR simulators, simulators with artificial eye models are considered to be very practical because they are easy to reproduce the characteristics of the real human eye and actual surgical instruments can be applied to them. There are available systems simulating various kinds of eye surgery [8–10]. However, these systems lack important anatomical details, such as the SC structure and the spherical shape of the eye, which is necessary for the glaucoma surgery simulation. Previously we succeeded in demonstrating the anterior eye model having TM and SC structure by the blow-molding fabrication [11]. In that fabrication method, several issues remained such as poor yields, limited types of materials, constraints on other design requirements, and so on.

In this research, we introduce a new concept of the glaucoma surgery simulator for MIGS. The concept is comprised of an anterior eye model with the SC structure and a fluidic circulatory system. It can mimic high IOP close to glaucoma. First, we define the requirements and target performances for the simulator and design the prototype. Next, we establish a fabrication method of the anterior eye model with TM and SC by molding and bonding flexible polymer materials, and assemble the prototype including the model. Using the prototype, we measure the pressure change before and after cleaving membrane mimicking TM. Finally, we demonstrate the availability by comparing experimental results and target performances.

## Materials and methods: Concept description

### Requirements for glaucoma surgery training

**Fig 1:**
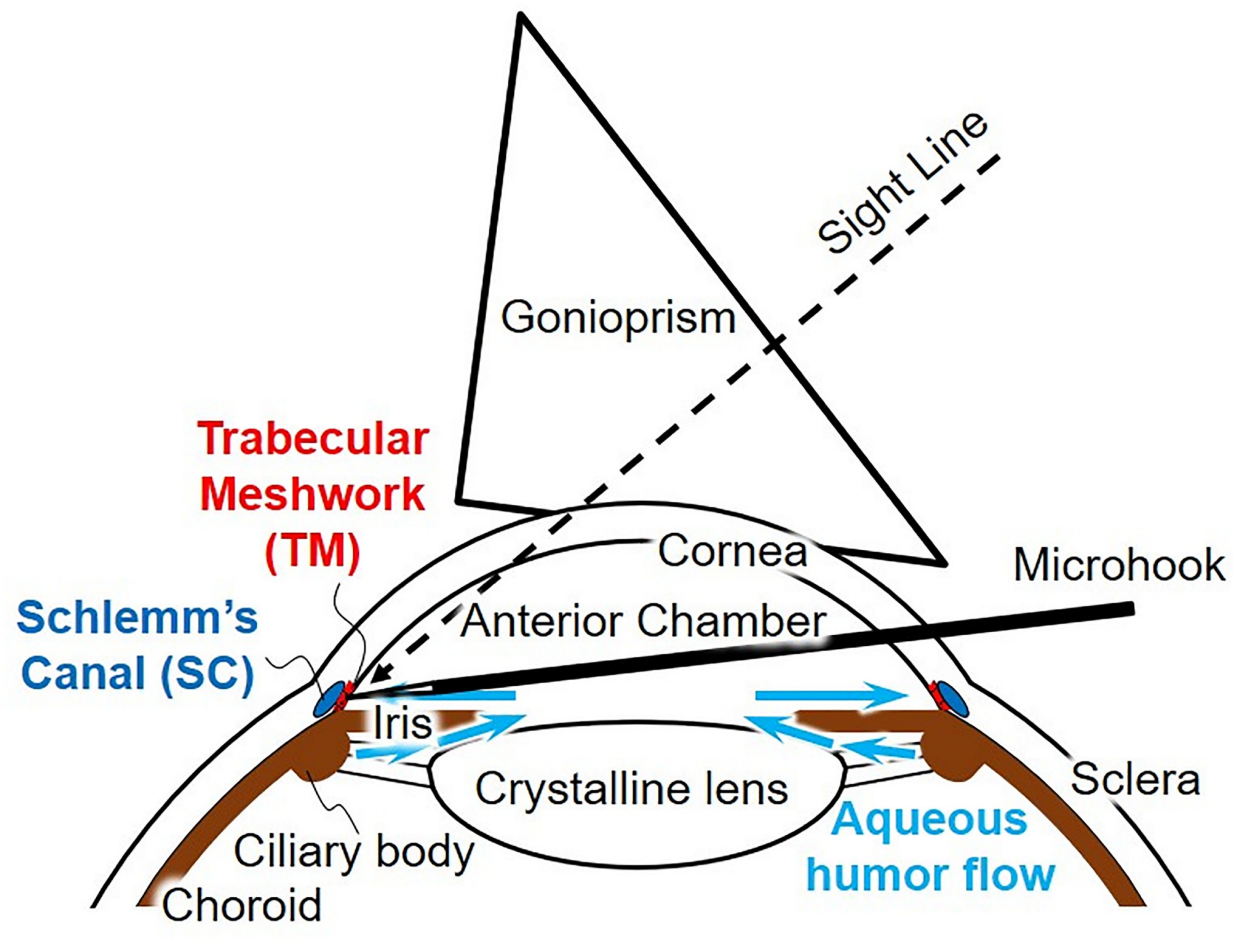
Anterior eye and minimally invasive glaucoma surgery (MIGS) procedure.

The structure of the anterior eye is shown in Fig 1 [12]. In the anterior eye, there is a Schlemm’s canal (SC) with an inner diameter of several hundred *μ*m [13] around the limbus cornea. The SC is covered with a thin membrane called trabecular meshwork (TM) with a thickness of 40–132 *μ*m [13]. Aqueous humor in the eye passes through the TM and is drained into the SC [12, 14–18]. The flow rate of aqueous humor is several *μ*L/min [12, 17]. In glaucoma, the IOP becomes higher than 21 mmHg (2.8 kPa) [17]. In “inside-out (ab interno) surgery” [3], the TM is incised using a microhook to reduce the IOP to the normal value of about 10 mmHg (1.3 kPa) (Fig 1) [17]. For the training of the surgery, we require an anterior eye model with the SC structure and a fluid circulating system that generates the pressure conditions.

### System configuration

**Fig 2:**
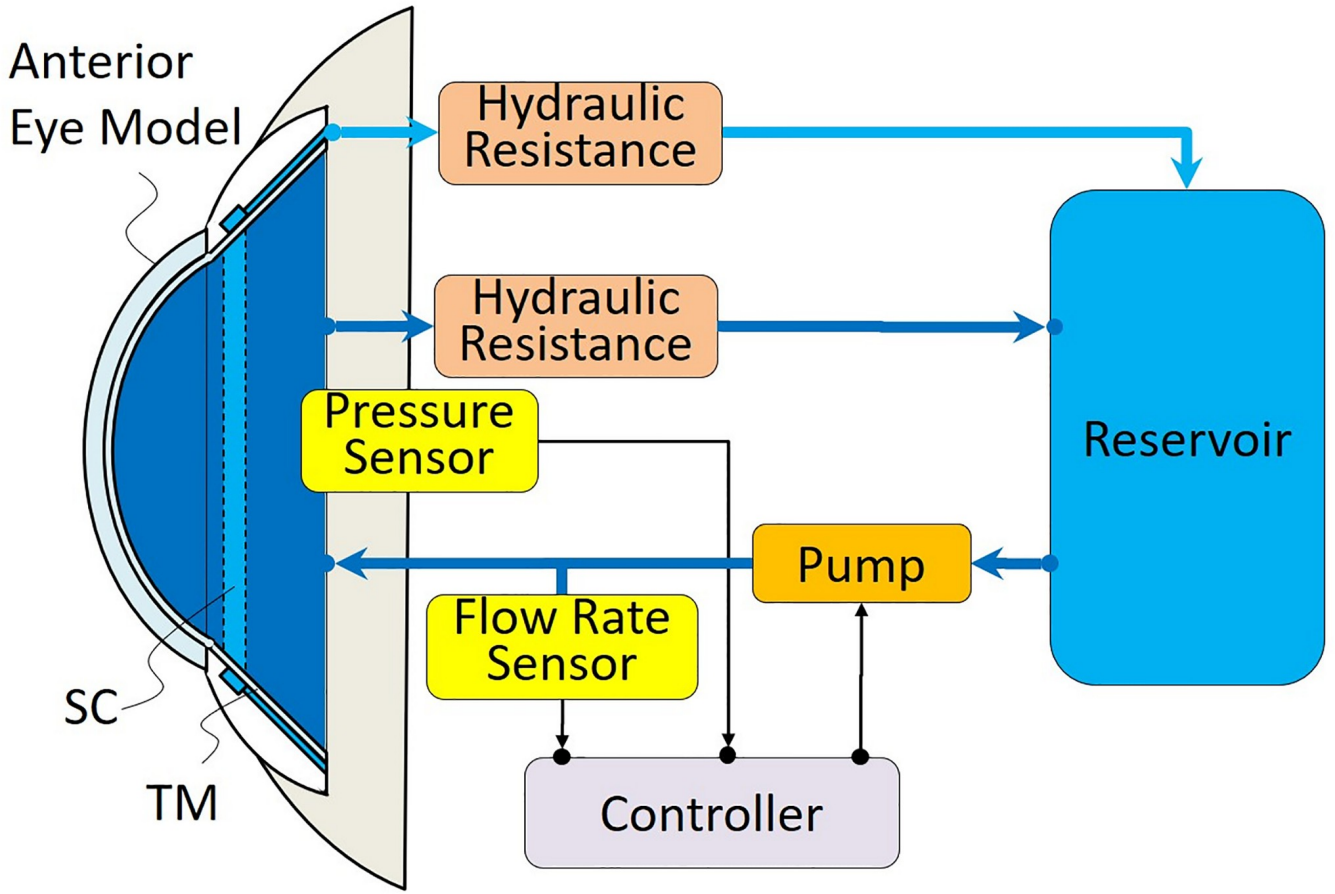
System configuration.

Fig 2 shows the system configuration that realizes this concept. It consists of an anterior eye model made of a flexible material such as polydimethylsiloxane (PDMS) and a fluid circulating system. The anterior eye model includes an intraocular (IO) space and an SC with a TM membrane. The system has IO and SC channels connected to a reservoir through hydraulic resistance pipes. The fluid circulating system uses a pump and controller to circulate fluid, such as water, in the IO channel. The IOP is measured by a pressure sensor placed in the IO space. The flow rate of aqueous humor is measured by a flow rate sensor placed between the pump and the anterior eye model. The pump is controlled so that the flow rate is 2–3 *μ*L/min. In the glaucoma state, the IOP is larger than about 3 kPa, depending on the constant flow rate and the hydraulic resistance between the IO space and the reservoir. When the TM is incised as the MIGS procedure, the two channels communicate with each other and the IOP drops to the normal value (about 1 kPa).

### Parametric design based on the hydraulic theoretical model

**Fig 3:**
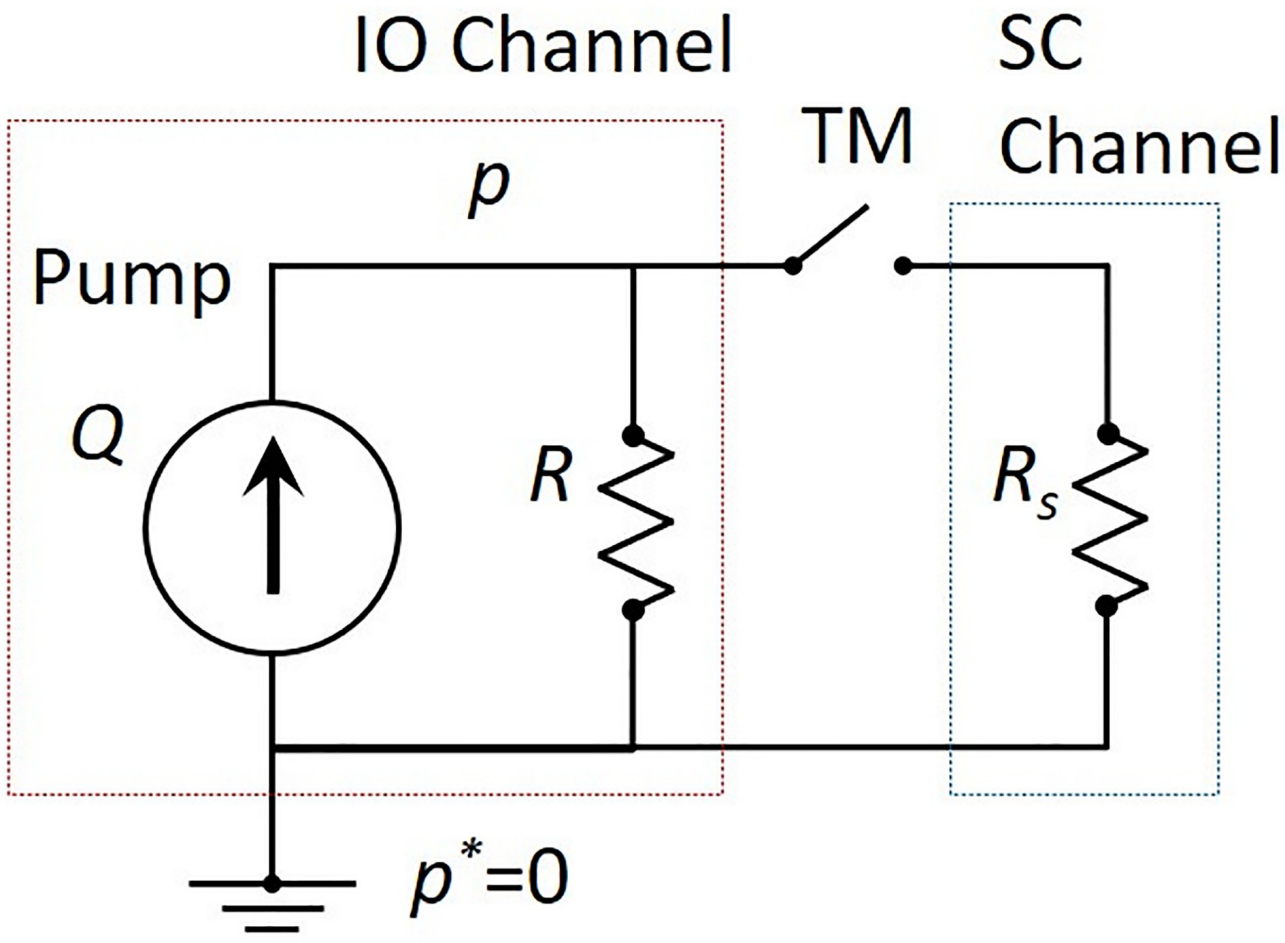
Hydraulic equivalent circuit.

Fig 3 shows the hydraulic equivalent circuit of this system. Hydraulic resistance of the pipe in the channels is generally described as follows [19]:

$$\begin{array}{ccc} R & = & \frac{128}{\pi }\frac{\eta L}{D^{4}} \end{array}$$ (1)

where *η*, *L*, *D* are the viscosity of the fluid, the length and internal diameter of the pipe. *D* is designed to be the minimum value for each channel to act as the maximum resistance.

Using (1), The IOP *p* before and after the MIGS is described as follows:

$$\begin{array}{ccc} p & = & \left\{ \begin{array}{ccccc} p^{-} & = & RQ & \cdots & \text{glaucoma}\\ p^{+} & = & \frac{R}{1+R/R_{s}}Q & \cdots & \text{after}\;\text{cleaving}\;\text{TM} \end{array}\right. \end{array}$$ (2)

where *Q* is the flow rate of the pump. Therefore the IOP drop is theoretically expected due to the decrease in the total hydraulic resistance after cleaving the TM membrane.

Table 1 shows the parameters designed to realize the human condition of glaucoma.

**Table 1:** Design parameters.

Meaning	Part	Symbol	Value (Units)	Comment
Viscosity	Fluid	*η*	1.000 (mPa s)	Water (293 K)
Flow rate	Pump	*Q*	3.3–5.0 × 10^−2^ (mm^3^/s)	= 2.0–3.0 (*μ*L/min)
Inner diameter	Pipe	*D*	63 (*μ*m)	-
Length	Pipe	*L*	35 (mm)	-
Hydraulic resistance	Pipe	*R*	91 (kPa s/mm^3^)	$=\frac{128\eta L}{\pi D^{4}}$
Hydraulic resistance	Pipe	*R*_*s*_	45 (kPa s/mm^3^)	*R*/2
IOP(Glaucoma)	Anterior Eye	*p* ^−^	> 2.8 (kPa)	= *RQ*
IOP(after cleaving TM)	Anterior Eye	*p* ^+^	1.3–2.8 (kPa)	$=\frac{R}{1+R/R_{s}}Q=RQ/3$

### Prototype design of glaucoma eye module

#### Glaucoma eye module

**Fig 4:**
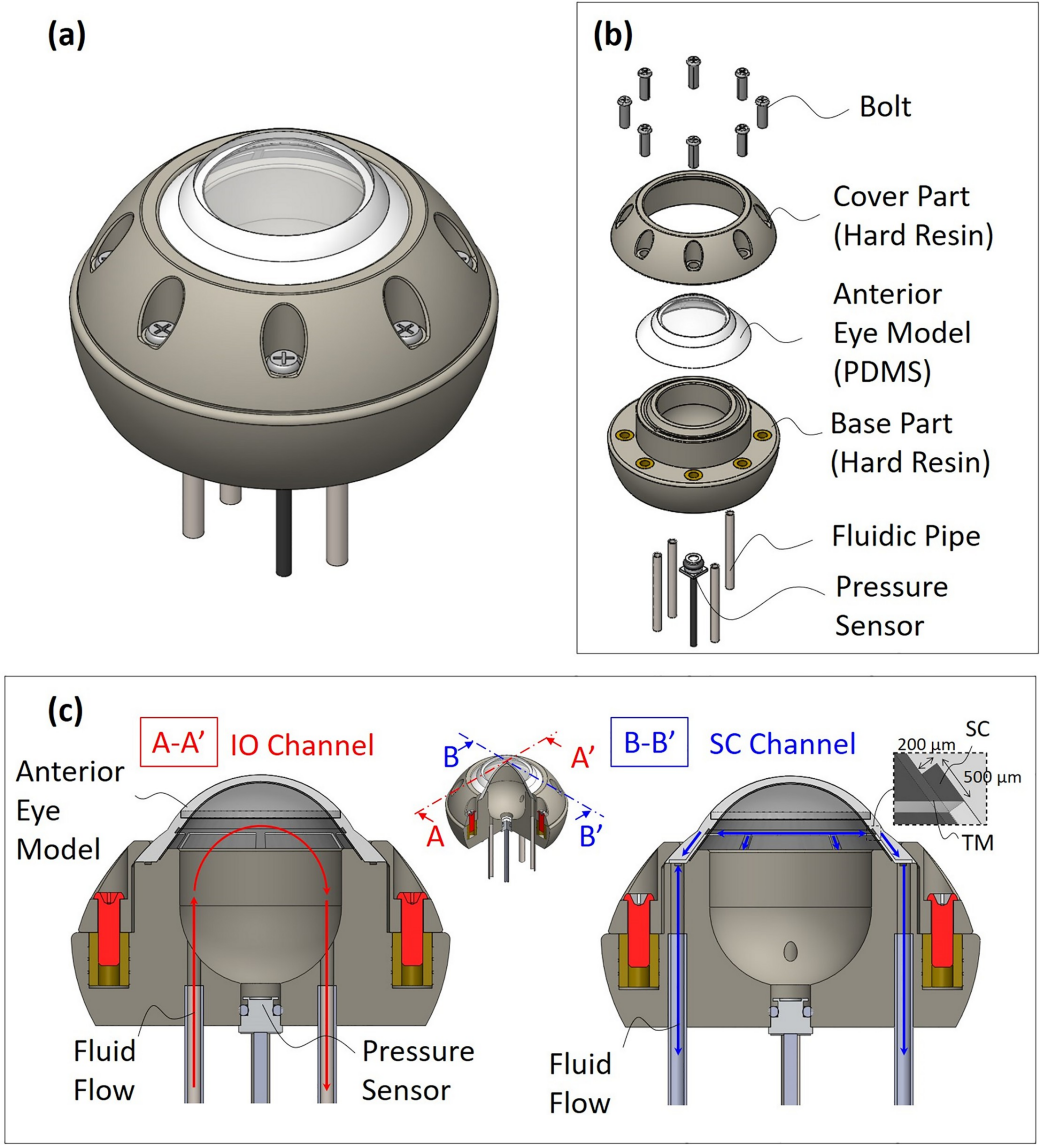
A glaucoma eye module design. (a) Assembled state. (b) Configuration. (c) Channel structure.

Fig 4 shows an eye module prototype of this concept. This module was assembled by sandwiching an anterior eye model made of PDMS from above and below with cover and base parts made of hard resin such as ABS. The cover and base parts were manufactured by 3D printing. Pipes and a pressure sensor can be attached to the base part.

The eye module assembled includes the IO and SC channels as shown in Fig 4(c). In addition, the glaucoma eye module can be mounted to the eye surgery simulator (bionic eye surgery evaluator: Bionic-EyE™ [20]) which was developed by our group.

#### Anterior eye model with Schlemm’s Canal (SC) structure

The anterior eye model is made of PDMS. PDMS has optical transparency and is often used for building artificial organ models [21–23]. Its elastic modulus of 1.3–3.0 MPa [24], very close to that of real sclera tissue (2.0 MPa) [25]. In addition, its optical transparency facilitates a clear visualization of the SC through the cornea using a goniolens during the surgery simulation [11].

**Fig 5:**
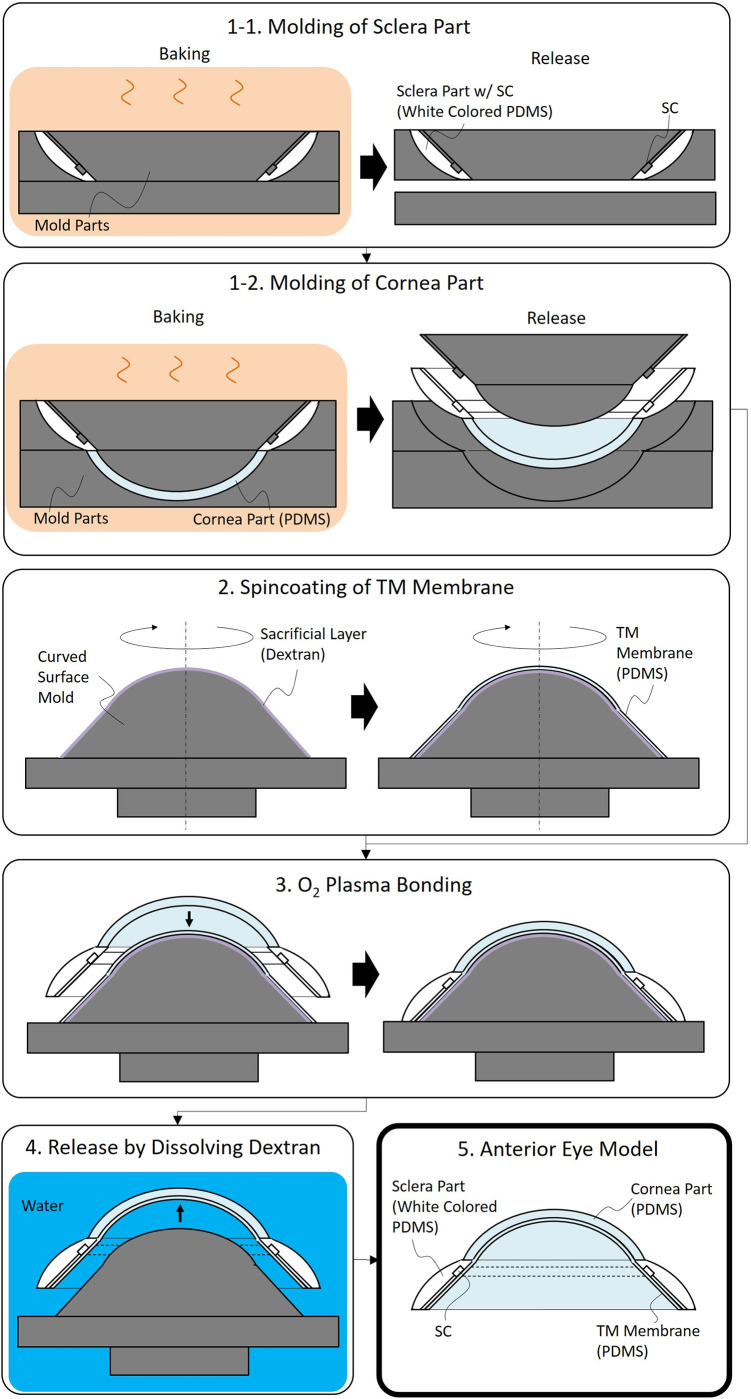
Fabrication process of the anterior eye model.

The anterior eye model was fabricated using the process as shown in Fig 5. First, the sclera and cornea parts were molded using PDMS as a material. By dividing the process into two steps, the only sclera part can be also made white-colored. The TM membrane was manufactured by spin-coating dextran as a water-soluble sacrificial layer [26] onto a curved-surface mold and then spin-coating uncured PDMS on the sacrificial layer. Next, those anterior parts and the TM membrane coated on the mold were bonded to each other just after being activated by the O_2_ plasma. Finally, the anterior eye model was released by dissolving the dextran layer in water.

## Results: Proof of concept

### Prototype

**Fig 6:**
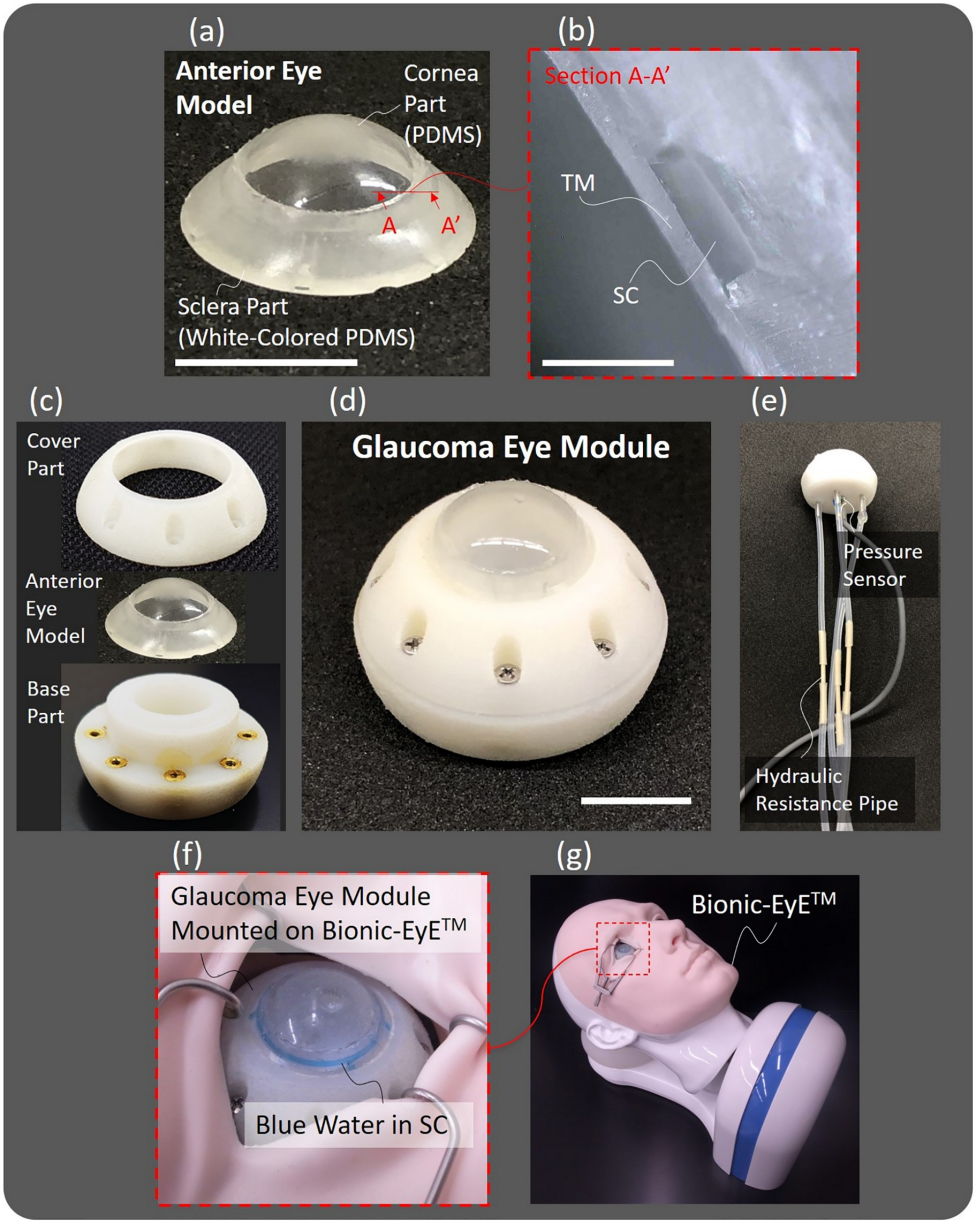
MIGS simulator prototype. (a) Fabricated anterior eye model. (b) A microscopic image of the section around the SC. A bar means 500 *μ*m. The TM thickness was 90 *μ*m and the cross-section widths of the SC were 170 and 550 *μ*m. (c) Configuration of glaucoma eye module. (d) Assembled glaucoma eye module. A bar means 10 mm. (e) Fluid/electrical components on the outside of the glaucoma eye module. (f) The glaucoma eye module in which blue water is injected into the SC. (g) The glaucoma eye module mounted on the Bionic-EyE™ [20].

Fig 6(a) shows a fabricated anterior eye model using the fabrication process in Fig 5. Fig 6(b) shows a microscopic image of the section around the SC. The cross-section widths of the SC were 170 and 550 *μ*m, which are close to those of the human eye [13]. The TM thickness was about 90 *μ*m under the condition that the rotation speed of the spin-coating was 4000 RPM. The thickness seems valid for the simulator because the human’s TM thickness is 40–132 *μ*m [13]. This process made it possible to manufacture this model with good yield.

The glaucoma eye module (Fig 6(d)) was assembled by sandwiching the anterior eye model between the cover and base parts (Fig 6(c)). Fig 6(e) shows fluid/electrical components on the outside of the module. The hydraulic resistance pipes made of PEEK were connected between the glaucoma eye module and the reservoir. The designed parameters *D*, *L*, *R*, *R*_*s*_ of the pipes are shown in Table 1. Those pipes function as dominant fluidic resistances *R*, *R*_*s*_ which mimic the gross aqueous outflow resistance composed of the trabecular and nontrabecular pathways [27].

The glaucoma eye module can be mounted to the Bionic-EyE™ [20] (Fig 6(g)). As shown in Fig 6(f), we confirmed that the SC structure was fabricated without any leaks by injecting blue-colored water into it.

### Minimally invasive glaucoma surgery (MIGS) training

**Fig 7:**
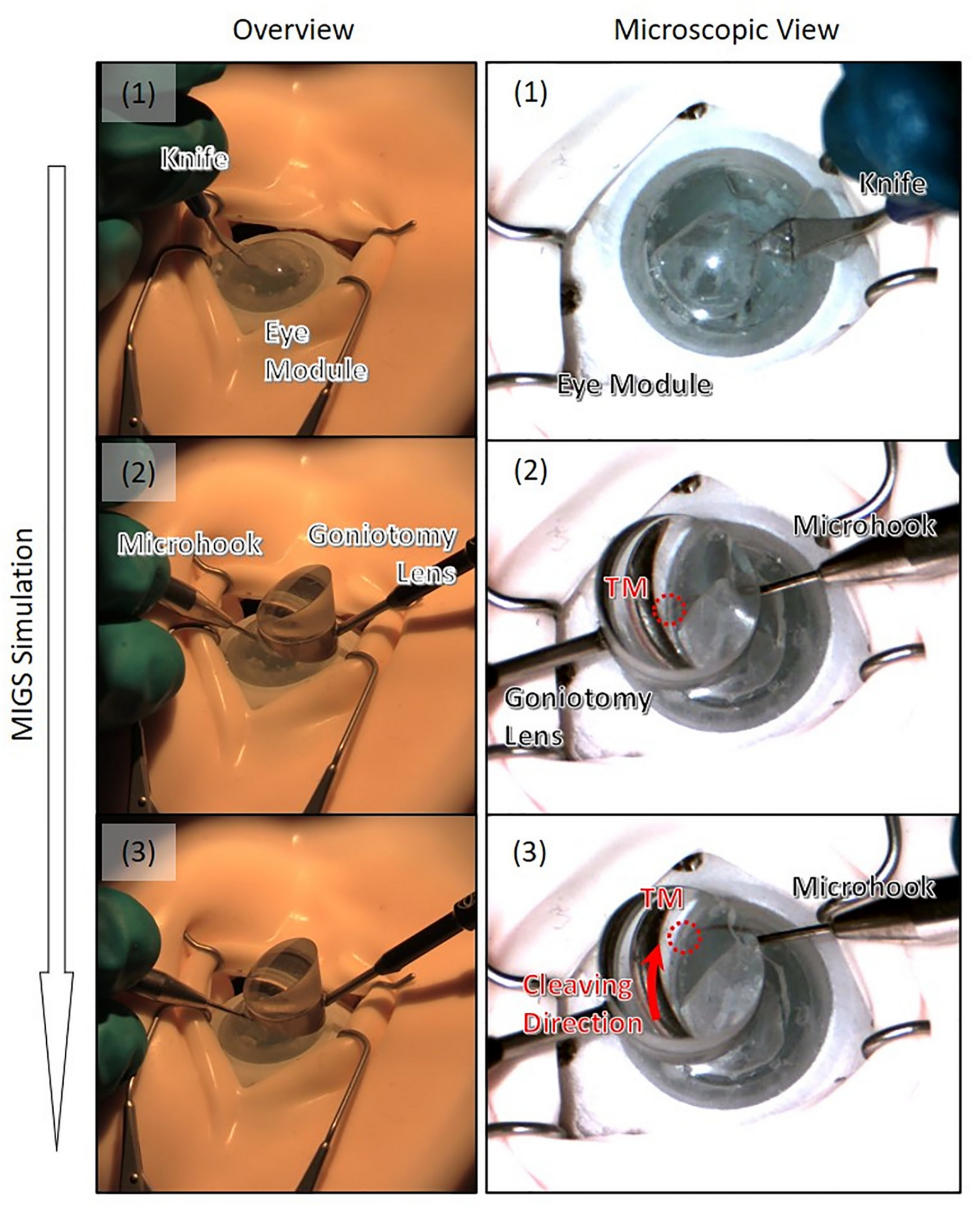
Snapshots of the MIGS procedure. (1) Making a small hole in the cornea by incising it. (2)(3) Cleaving the TM membrane in the eye module.

Using the glaucoma eye module with the fluid circulatory system, we demonstrated the MIGS training. We did the procedure similar to the “inside-out (ab interno) surgery” as one of the MIGS procedures. Fig 7 shows the snapshots. First, the IO channel was filled with water by turning on the pump, and at that time, this module should be in the glaucoma state under the above design. Next, a small hole was made in the cornea by incising it with a medical knife (Fig 7(1)). Observing the TM through a microscope and a goniotomy lens, we were able to cleave the TM with the microhook through the hole (Fig 7(2) and 7(3)). Finally, we closed the hole by gluing it.

### Intraocular pressure (IOP) and aqueous flow rate

**Fig 8:**
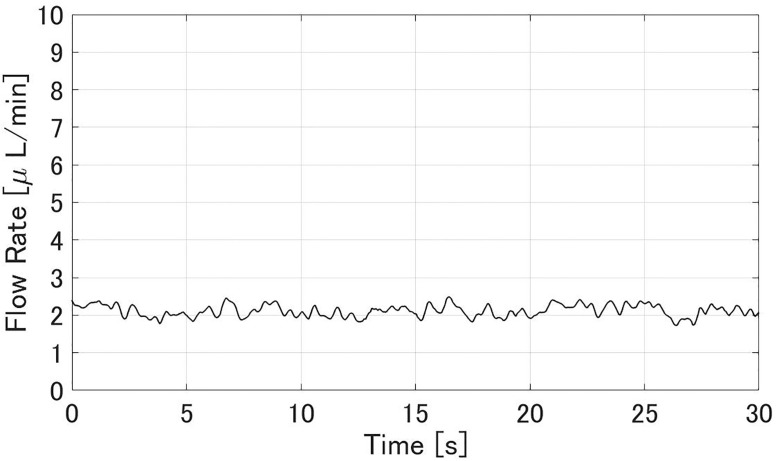
An experimental result of the flow rate *Q* when simulating glaucoma. The sampling interval was 0.07 s. *Q* was 2.1±0.4 *μ*L/min.

**Fig 9:**
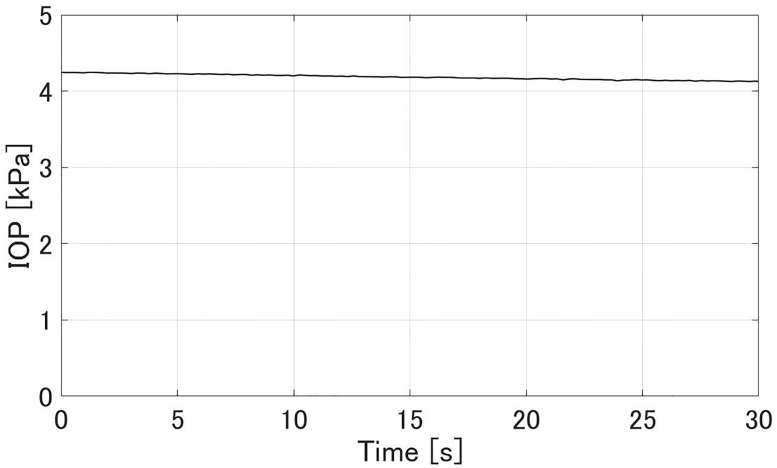
An experimental result of the IOP *p*^−^ when simulating glaucoma. The sampling interval was 0.26 s. *p*^−^ was 4.18±0.06 kPa.

**Fig 10:**
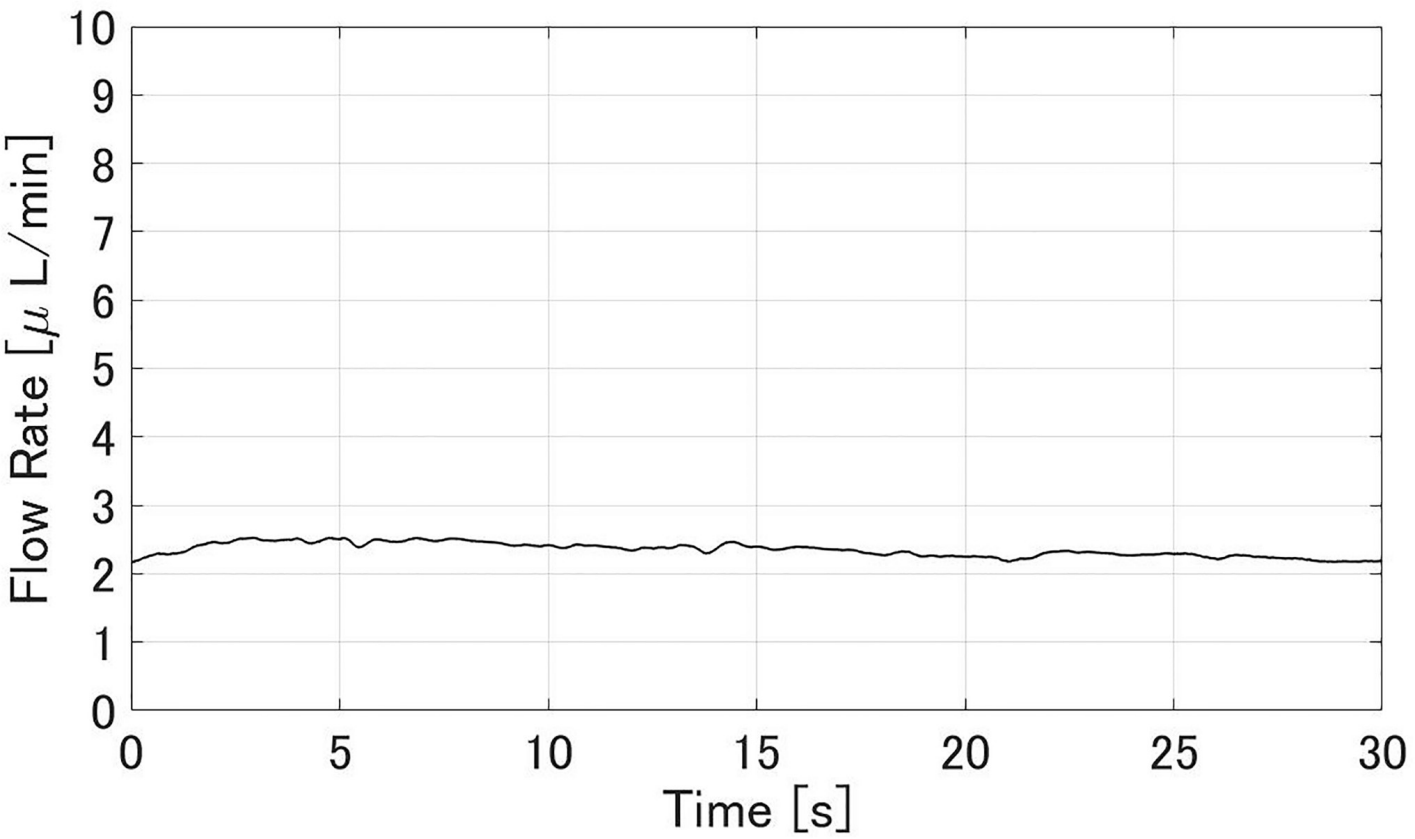
An experimental result of the flow rate *Q* after cleaving the TM membrane and adhesively closing the small incised part in the cornea. The sampling interval was 0.07 s. *Q* was 2.4±0.2 *μ*L/min.

**Fig 11:**
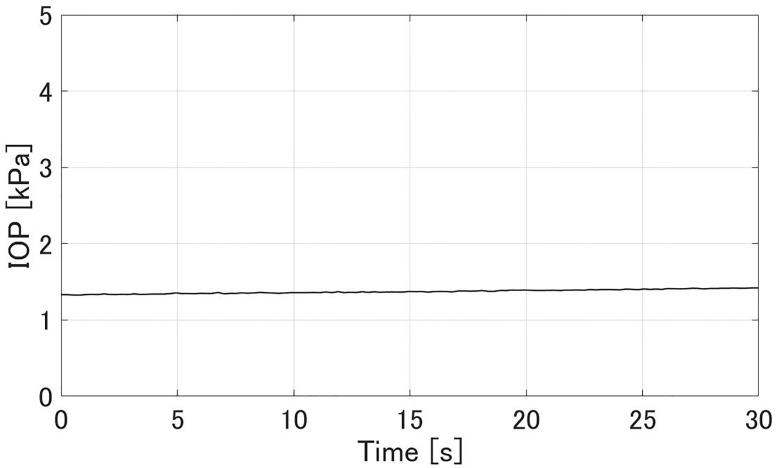
An experimental result of the IOP *p*^+^ after cleaving the TM membrane and adhesively closing the small incised part in the cornea. The sampling interval was 0.26 s. *p*^+^ was 1.37±0.05 kPa.

Figs 8 and 9 show experimental results of the aqueous flow rate *Q* and IOP *p*^−^ in the glaucoma state, respectively. The flow rate *Q* was stable at 2.1±0.4 *μ*L/min. The IOP *p*^−^ was 4.18±0.06 kPa. Figs 10 and 11 show experimental results of the *Q* and *p*^+^ after all MIGS procedures in Fig 7, respectively. The flow rate *Q* was stable at 2.4±0.2 *μ*L/min, similar to the value in glaucoma. The IOP *p*^+^ was reduced to 1.37±0.05 kPa compared to the *p*^−^.

## Discussion

As shown in Fig 7, we were able to demonstrate the MIGS procedures using the glaucoma eye module, so it is considered that this module can be used for the MIGS training.

In the glaucoma state (Figs 8 and 9), the aqueous flow rate *Q* was in the range 2–3 *μ*L/min and IOP *p*^−^ was larger than 2.8 kPa. After the MIGS procedures (Figs 10 and 11), *Q* stayed in that range and *p*^+^ was reduced to about *p*^−^/3. Those results meet the design in Table 1. Therefore the IOP drop by the MIGS procedure was quantitatively demonstrated similar to the human condition by using the glaucoma eye module.

There were some errors in the total hydraulic resistances *R*, *R*_*s*_ between design values and experimental results. The resistances *R* = *p*^−^/*Q*, *R*_*s*_ = *p*^+^/*Q* and those errors Δ*R*/*R*, Δ*R*_*s*_/*R*_*s*_ were experimentally estimated as 119 kPa s/mm^3^, 35 kPa s/mm^3^, 31%, and −16%, respectively. The main reason is considered to be size variations of the hydraulic resistance pipes. From Eq (1), the resistance errors are described due to the variations Δ*L*,Δ*D* as follows:

$$\Delta R_{L}/R=\Delta L/L,$$ (3)

$$\Delta R_{D}/R={\left(1+\Delta D/D\right)}^{-4}-1≃-4\Delta D/D.$$ (4)

From Eqs (3) and (4), Δ*D*/*D* is more sensitive to the resistance errors than Δ*L*/*L*. Assuming Δ*L* = 1 mm (Δ*L*/*L* = 2.9%) and Δ*D* = −4 *μ*m (Δ*D*/*D* = 6.3%), the resistance error Δ*R*/*R* is estimated as 34%. Another reason might be the pressure difference by bubbles trapped in the pipe [19]. However, the flow velocity in the pipe is estimated to be 11 mm/s, and the assumed bubble should pass through the pipe in about 3 s. Therefore, the bubble pressure is unlikely to affect the steady resistance errors.

## Conclusion

In this research, we designed, manufactured, and evaluated the glaucoma eye module with a fluid circulatory system as the artificial simulator for MIGS surgery. Especially, we quantitatively demonstrated the IOP drop similar to the human condition before and after the MIGS procedure by using the simulator.

In the near future, we will realize a more practical glaucoma surgery simulator with a human-like appearance and characteristics by improving it based on the evaluation results of ophthalmologists. This simulator is also expected to be used for evaluations and developments of new MIGS instruments and ophthalmic surgery robots in addition to the surgical training of ophthalmologists.

## Supporting information

S1 Video MIGS procedure. This video describes the MIGS procedure experiment in the paper, which is a real-time scale (that is, 1x speed). This video corresponds to Fig 7 in the paper.
(MP4)
